# Lymphocyte Subpopulations in Lymph Nodes and Peripheral Blood: A Comparison between Patients with Stable Angina and Acute Coronary Syndrome

**DOI:** 10.1371/journal.pone.0032691

**Published:** 2012-03-01

**Authors:** Karin Backteman, Carina Andersson, Lars-Göran Dahlin, Jan Ernerudh, Lena Jonasson

**Affiliations:** 1 Division of Clinical Immunology, Department of Clinical and Experimental Medicine, Faculty of Health Sciences, Linköping University, Department of Clinical Immunology and Transfusion Medicine, County Council of Östergötland, Linköping, Sweden; 2 Division of Clinical Immunology, Department of Clinical Immunology and Transfusion Medicine, County Council of Östergötland, Linköping, Sweden; 3 Department of Thoracic Surgery, County Council of Östergötland, Linköping, Sweden; 4 Division of Cardiovascular Medicine, Department of Medical and Health Sciences, Faculty of Health Sciences, Linköping University, Department of Cardiology, County Council of Östergötland, Linköping, Sweden; University of Nebraska Medical Center, United States of America

## Abstract

**Objective:**

Atherosclerosis is characterized by a chronic inflammatory response involving activated T cells and impairment of natural killer (NK) cells. An increased T cell activity has been associated with plaque instability and risk of acute cardiac events. Lymphocyte analyses in blood are widely used to evaluate the immune status. However, peripheral blood contains only a minor proportion of lymphocytes. In this study, we hypothesized that thoracic lymph nodes from patients with stable angina (SA) and acute coronary syndrome (ACS) might add information to peripheral blood analyses.

**Methods:**

Peripheral blood and lymph nodes were collected during coronary by-pass surgery in 13 patients with SA and 13 patients with ACS. Lymphocyte subpopulations were assessed by flow cytometry using antibodies against CD3, CD4, CD8, CD19, CD16/56, CD25, Foxp3, CD69, HLA-DR, IL-18 receptor (R) and CCR4.

**Results:**

Lymph nodes revealed a lymphocyte subpopulation profile substantially differing from that in blood including a higher proportion of B cells, lower proportions of CD8^+^ T cells and NK cells and a 2-fold higher CD4/CD8 ratio. CD4^+^CD69^+^ cells as well as Foxp3^+^ regulatory T cells were markedly enriched in lymph nodes (p<0.001) while T helper 1-like (CD4^+^IL-18R+) cells were more frequent in blood (p<0.001). The only significant differences between ACS and SA patients involved NK cells that were reduced in the ACS group. However, despite being reduced, the NK cell fraction in ACS patients contained a significantly higher proportion of IL-18R^+^ cells compared with SA patients (p<0.05).

**Conclusion:**

There were several differences in lymphocyte subpopulations between blood and lymph nodes. However, the lymphocyte perturbations in peripheral blood of ACS patients compared with SA patients were not mirrored in lymph nodes. The findings indicate that lymph node analyses in multivessel coronary artery disease may not reveal any major changes in the immune response that are not detectable in blood.

## Introduction

Atherosclerosis is characterized by a chronic inflammatory response and the immunological activity in the atherosclerotic plaque is considered an important determinant in the disease process. The transition from a stable plaque to an unstable rupture-prone plaque has been associated with an increased number of intra-plaque T cells exhibiting early signs of activation [1], [2]. There is a dominant expression of T helper (Th)1 cytokines, such as interferon (IFN)-gamma in advanced human carotid and femoral lesions [3] and recently, IFN-gamma was found to be highly produced by CD4^+^ T cells within human coronary plaques [4].

Previous reports have also shown that the adaptive immune response in peripheral blood differs when comparing chronic and acute manifestations of coronary artery disease. Compared with patients with stable angina (SA), patients with acute coronary syndrome (ACS) exhibit increased numbers of circulating CD4^+^ T cells with phenotypical characteristics of Th1 as well as enhanced expression of both early and late activation markers [5], [6], [7], [8], [9], [10], [11]. Interestingly, Steppich and coworkers found that the activation of peripheral T cells in ACS did not correlate with markers of myocardial damage suggesting that T cell activation might have been a plaque destabilizing factor preceding the acute cardiac event [11]. However, a few studies have failed to demonstrate elevations in T cell activation markers or serologic evidence for increased IFN-gamma production in ACS compared with SA [12], [13]. Other studies have reported differences between SA and ACS regarding other lymphocyte populations in peripheral blood, such as reductions in natural killer (NK) cells and regulatory T (Treg) cells in patients with ACS [6], [14], [15]. Moreover, a reduction of Treg cells in ACS has been found to be consistent with an expansion of Th1 cells [6].

One relevant question is to what extent the lymphocyte changes in peripheral blood reflect the immune response in atherosclerotic lesions. Probably only 2% of the lymphocyte pool is present in the peripheral blood [16]. Lymphocytes recirculate continuously between the blood and lymphoid organs. In the lymph nodes, where 40% of the total amount of lymphocytes is assumed to reside, the adaptive immune response is both initiated and regulated. The investigation of regional lymph nodes may thus have the potential to provide new insight into the immune activation of coronary artery disease. In the present study, we examined the distribution of lymphocyte subpopulations in thoracic lymph nodes from patients with SA and ACS hypothesizing that lymph node analyses reveal changes in the immune response that are not detectable in peripheral blood.

## Methods

### Ethics Statement

Written informed consent was obtained from study participants and the study protocol was approved by the regional ethics committee at Linköping University. The study was conducted in accordance with ethical guidelines of Declaration of Helsinki.

### Selection of subjects and collection of samples

We included 26 patients with multivessel coronary artery disease that were referred for coronary artery bypass surgery (CABG) at the Department of Thoracic Surgery, University Hospital, Linköping during 2008–2010. Thirteen SA patients underwent elective surgery due to effort angina class II or III according to Canadian Cardiovascular Society Classification and without any worsening of symptoms the latest 3 months. Thirteen ACS patients underwent surgery soon after a non-ST elevation myocardial infarction, *i.e.* within 14 (11–19) days. In combination with the clinical presentation, the diagnosis of non-ST elevation myocardial infarction was based on typical ECG-changes (ST-T segment depression and/or T-wave inversion) and elevated troponin levels [17]. Patients were excluded if they had severe heart failure, renal or hepatic disease, neoplastic disease, immunologic disorders or treatment with immunosuppressive agents.

During harvesting of the internal mammary artery for subsequent use as an arterial graft to a coronary vessel, the attentive surgeon occasionally (in approximately 20% of cases) encounters lymph nodes in the adjacent tissues of the arterial pedicle. Such lymph nodes are normally overlooked or trimmed away from the pedicle and then discarded. In the present study we collected this surgical waste material prior to administration of heparin and extracorporeal circulation, *i.e.* in the initial phase of surgery. Samples of venous peripheral blood were drawn into EDTA tubes. Blood and lymph nodes were transported immediately after collection to the laboratory for analysis.

We also collected blood from 26 controls (20 men, mean age 67 years, range 49–77 and 6 women, mean age 69 years, range 62–76) randomly selected from the population register. They were all clinically healthy without taking any medication and with normal routine laboratory tests.

### Cell populations

Lymphocyte subpopulations from peripheral blood and lymph nodes were measured by 6- or 7-color combinations. Cells were stained with the following monoclonal antibodies against surface markers: CD3-FITC (clone SK7), HLA-DR-FITC (clone L234), CD56-PE (clone NCAM16.2), CD3-PerCP (clone SK7), CD4-PerCP (clone SK7), CD45-PerCP (clone 2D1), CD4-PE Cy7 (clone SK3), CD56-PE Cy7 (clone NCAM16.2), CD69-PE Cy7 (clone FN50), CCR4-PE Cy7 (clone 1G1), CD19-APC (clone J25C1), CD25-APC (clone 2A3), CD56-APC (clone NCAM16.2), CD8-APC H7 (clone SK1), CD3-APC H7 (cloneSK7), CD3-Pacific Blue (clone UCHT1), all from BD Biosciences, San José, CA, US. Anti-interleukin (IL)-18 receptor (R)α-PE (clone 70625) was purchased from R&D systems, Minneapolis, MN, US, and anti-Foxp3-PE (clone PCH101) from eBioscience, San Diego, CA, US.

### Lymph node preparation

A single-cell suspension was prepared from the lymph node within 1 hour after extirpation. The unfixed lymph node was first cut into 3–5 mm pieces and then disaggregated mechanically into single-cells by rotating knifes in a Medimachine (DAKO, Hamburg, Germany). The cells were resuspended in phosphate buffered saline and then filtrated with a 50 µm Filcons filter. The cell concentration was adjusted to 20×10^6^/mL.

Fifty µL of the EDTA blood or lymph node suspension were added to appropriate amounts of each antibody and incubated for 15 minutes in the dark at room temperature. Thereafter, erythrocytes were lysed with 450 µL FACS™ Lysing Solution (BD Biosciences) for 15 minutes at room temperature in darkness. After erythrocyte lysis, cells were washed with phosphate-buffered saline with 2% human serum albumin before flow cytometry analysis.

For intracellular staining with Foxp3, 100 µL of the EDTA blood or lymph node suspension were permabilized according to the manufacturer's instructions. In brief; cells were incubated with appropriate amounts of HLA-DR, CD4, CD69, CD25 and CD3 and erythrocytes lysed. Fixation/permeabilization buffer (eBioscience) was added and followed by incubation for 30 min at 4°C in darkness. The antibody against Foxp3 was added followed by incubation for 30 min at 4°C in darkness. Finally the cells were washed, resuspended in washing buffer and immediately analysed by flow cytometry.

### Flow cytometry gating and analysis

The analyses of lymphocytes subpopulations were performed on a FACSCanto II (BD Biosciences) equipped with 3 lasers, blue 488 nm, a red 633 nm and a violet 405 nm. Control of the instrument settings was done daily with 7-color Setup Beads™ with FACSDiva™ software or Cytometer Setup and Tracking beads™ (BD Biosciences) with Cytometer Setup and Tracking™ software according to the standard procedure. A median of 23 500 (range 6,200–90,300) peripheral lymphocytes and 95 000 (range 4,800–116,700) lymph node cells were analysed. Data analyses were performed with FACSDiva™ 6.1.2 software (BD Biosciences).

**Figure 1:**
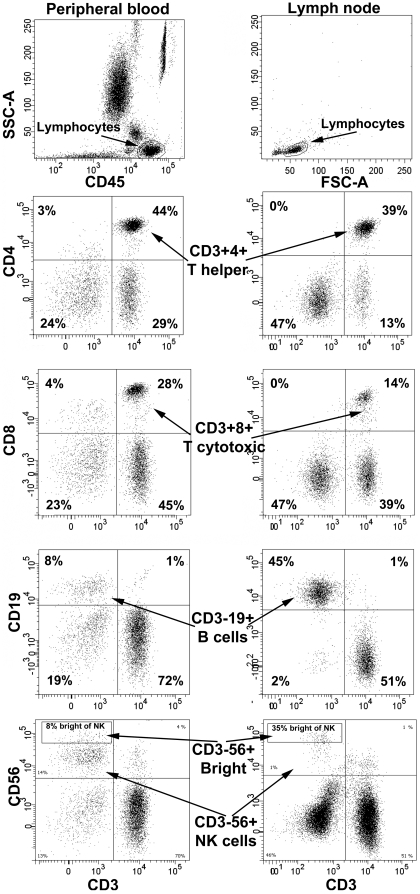
Gating strategies for major lymphocyte populations. Gating strategies for major lymphocyte populations in peripheral blood and lymph node respectively. Values are given as percent of lymphocytes. For CD56^bright^ subpopulation value is presented as percent of CD56. Examples from one ACS (acute coronary syndrome) patient shows dot plots representative for both SA (stable angina) and ACS patients.

**Figure 2:**
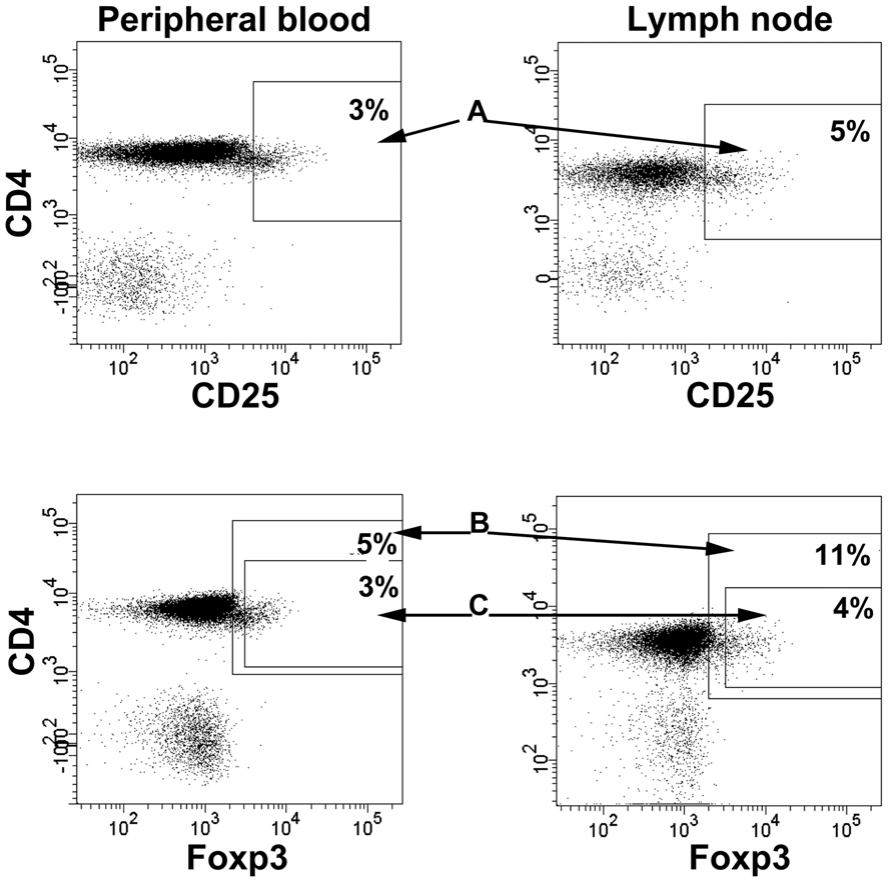
Gating strategies for regulatory T cells. Different strategies for gating regulatory T cells in peripheral blood and lymph node respectively. Panel A; Regulatory T cells is based on CD3 expression (not shown) ,slightly lower (dim) CD4 expression and high (bright) expression of CD25, termed CD4^dim^/CD25^bright^ [18] Panel B; Regulatory T cells based on CD3 expression (not shown), Foxp3 expression on CD4 population and with limit of the CD8 population ( = old gating strategy). Panel C; Regulatory T cells is based on CD3 expression (not shown), high (bright) expression of CD25 (not shown), slightly lower (dim) CD4 expression and high Foxp3 expression. Values are given as percent CD3^+^4^+^. Examples from one ACS (acute coronary syndrome) patient shows dot plots representative for both SA (stable angina) and ACS patients.

Lymphocytes were identified by CD45/forward scatter or side scatter/forward scatter. Analysis of subpopulations was based on contour gating. Gating strategies for the major lymphocyte subpopulations in peripheral blood and lymph nodes are presented in Figure 1. T reg cells were gated on the basis of CD3 expression and CD4 expression that was slightly lower (dim) than the remaining CD4 population, in combination with higher (bright) expression of CD25 and Foxp3, as previously described [18] (see Figure 2).

The flow cytometry laboratory gained accreditation status in 1996 according to ISO/IEC 17025 standards and we participate and perform well in external quality programs for immune phenotyping (UK-Nequas and Equalis).

### Statistical methods

Statistical analyses were done in SPSS® 17.0. Differences within groups were analyzed using Wilcoxon signed-rank test while the Mann-Whitney U test was used for between-group analyses. Correlation analysis was performed using Spearman rank correlation test. Statistical significances were set at a two-tailed p-value of <0.05. Values are given as medians and inter-quartile range.

## Results

Patients' characteristics are listed in Table 1. All patients were treated with low-dose aspirin and various combinations of nitrates, beta-blockers and/or calcium antagonists. Ten of 13 SA patients and 4 of 13 ACS patients were on long-term treatment, i.e. >2 months, with statin. The remaining 9 ACS patients had received statin therapy after admission for the index event, i.e. within <1 month. In addition, low-molecular weight heparin had been introduced on admission and continued until the day before surgery in all ACS patients. Four of 13 SA patients had a history of prior myocardial infarction while one of 13 ACS patients had a history of myocardial infarction before the index event.

**Table 1:** Characteristics of SA and ACS patients.

	SA(n = 13)	ACS(n = 13)
Age, years	74 (61–84)	66 (49–82)
Men (n)	10	10
Hypertension (n)	6	9
Diabetes (n)	2	6
Smokers, current (n)	0	1
Statin treatment (n)		
long term	10	4
<1 month	3	9
Serum creatinine, µmol/L.	94 (78–141)	87 (64–117)

There were no significant differences in age or serum creatinine between SA and ACS. Values are given as median (range).

The distributions of the major lymphocyte subpopulations in peripheral blood compared with lymph nodes are presented in Table 2. CD3^+^ T cells constituted the majority of lymphocytes in both blood and lymph nodes. In ACS patients, the proportion of CD3^+^ T cells was significantly higher in blood than in lymph nodes and so was the proportion of CD8^+^ T cells. In SA patients, these differences showed the same pattern though less prominent reaching significance only for CD8+ T cells. In contrast, the proportions of CD4^+^ T cells were similar in blood and lymph nodes resulting in a CD4/CD8 ratio that was around 2-fold higher in lymph nodes. B cells (CD3^−^CD19^+^) were abundantly present in lymph nodes. NK cells (CD3^−^CD56^+^), on the other hand, exhibited low numbers in lymph nodes compared with blood. Among NK cells, the CD56^bright^ subset was more frequently found in lymph nodes than in peripheral blood although the CD56^dim^ subset still dominated in both blood and lymph nodes. There were no significant differences in T cells, B cells, CD56^bright^ or CD56^dim^ NK cell subsets between SA and ACS patients in either blood or lymph nodes. However, in peripheral blood of ACS patients the proportion of all NK cells (CD3^−^CD56^+^) was significantly reduced compared with SA patients.

**Table 2:** Distribution of major lymphocyte subpopulations.

		SA patients (n = 13)	ACS patients (n = 13)	p
**CD3^+^ % of lymphocytes**	blood	67 (64–72)	72 (66–79)	ns
	lymph node	60 (48–67)§	55 (45–60)**	ns
**CD3^+^4^+^ % of lymphocytes**	blood	40 (27–51)	42 (35–46)	ns
	lymph node	43 (35–53)	41 (37–47)	ns
**CD3^+^8^+^ % of lymphocytes**	blood	24 (17–35)	29 (20–34)	ns
	lymph node	15 (9.3–21)*	14 (10–17)**	ns
**CD4/CD8 ratio**	blood	1.5 (1.0–3.2)	1.4 (1.2–1.9)	ns
	lymph node	2.8 (2.5–3.8)*	3.1 (2.7–4.0)**	ns
**CD3^−^19^+^ % of lymphocytes**	blood	7.0 (5.6–11)	11 (6.9–13)	ns
	lymph node	39 (30–50)**	43 (38–53)**	ns
**CD3^−^56^+^ % of lymphocytes**	blood	20 (12–23)	12 (9.4–19)	<0.05
	lymph node	1.6 (1.2–2.0)**	1.5 (1.3–2.4)**	ns
**CD3^−^56^bright^ % of CD3−56+**	blood	4.6 (3.2–8.1)	6.5 (5.1–11)	ns
	lymph node	31 (21–55)**	34 (13–39)**	ns
**CD3^−^56^dim^ % of CD3−56+**	blood	96 (93–98)	94 (90–96)	ns
	lymph node	72 (48–82)**	69 (60–86)**	ns

Values are given as median (inter-quartile range).

Significant differences between patients with stable angina (SA) and acute coronary syndrome (ACS) were found only in the CD3^−^56^+^ population in blood, shown in the column to the right.

Comparing blood and lymph nodes, there were significant differences in all populations except CD3^+^4^+^. Values * p<0.05, ** p<0.01 refer to differences between lymph nodes and blood. Value § p = 0.07 showed similar pattern for SA between lymph nodes and blood as for ACS.

As shown in Table 3, the proportions of CD4^+^ T cells expressing the early activation marker CD69^+^ were few in peripheral blood but enriched in lymph nodes. The proportions of CD4^+^ and CD8^+^ T cells expressing the late activation marker HLA-DR were similar in blood and lymph nodes. No differences were seen between SA and ACS patients regarding early or late activation markers in either blood or lymph nodes (Figure S1).

**Table 3:** Distribution of T cell activation markers and regulatory T cells.

		SA patients ( = 13)	ACS patients ( = 13)	p
**CD3^+^4^+^69^+^ % of CD3^+^4^+^**	blood	1.2 (0.9–1.8)	1.0 (0.9–1.8)	ns
	lymph node	40 (38–50)**	49 (41–58)**	ns
**CD3^+^4^+^HLA/DR^+^ % of CD3^+^4^+^**	blood	14 (7.4–18)	10 (7.5–13)	ns
	lymph node	13 (10–17)	13 (9.9–16)	ns
**CD3^+^8^+^HLA/DR^+^ % of CD3^+^8^+^**	blood	37 (28–43)	32 (17–60)	ns
	lymph node	32 (25–49)	35 (22–54)	ns
**CD3^+^4^dim^25^bright^ % of CD3^+^4^+^**	blood	2.9 (2.4–4.8)	3.0 (2.2–4.7)	ns
	lymph node	4.9 (3.7–6.6)	3.8 (2.0–8.0)	ns
**CD3^+^4^dim^ 25^bright^ Foxp3^+^ % of CD3^+^4^+^**	blood	2.0 (1.5–3.0)	2.1 (1.3–2.8)	ns
	lymph node	4.5 (2.8–5.3)**	3.6 (2.1–6.3)**	ns
**CD3^+^4^+^ Foxp3^+^ % of CD3^+^4^+^**	blood	5.2 (3.1–5.6)	4.5 (3.0–5.1)	ns
	lymph node	15 (10–17)**	17 (11–19)**	ns

Values are given as median (interquartile range).

No significant differences were noted between patients with stable angina (SA) and acute coronary syndrome (ACS). Comparing blood and lymph nodes, there were significant differences in CD3^+^4^+^69^+^ and T reg populations. Values ** p<0.01 refer to differences between lymph nodes and blood.

The proportions of Treg cells are presented as either CD4^dim^25^bright^, CD4^dim^25^bright^Foxp3^+^ or CD4^+^Foxp3^+^ (Figure 2, Table 3). Their proportions in peripheral blood were low and around 2-fold increased in lymph nodes. There were no differences in Treg distributions between SA and ACS patients.

**Figure 3:**
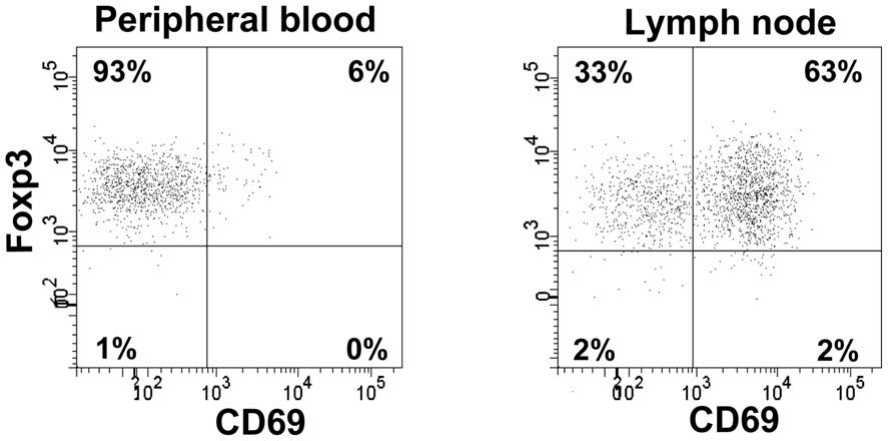
Gating strategy for Foxp3 and CD69. Gating strategy for Foxp3 and CD69 distribution on regulatory T cells in peripheral blood and lymph node respectively. The CD69/Foxp3 subset is based on CD4^dim^/CD25^bright^ (Fig. 2A). Values are given as percent CD4^dim^/CD25 bright. Examples from one ACS (acute coronary syndrome) patient shows dot plots representative for both SA (stable angina) and ACS patients.

The distribution of Foxp3 and CD69 in Treg cells (CD4^dim^25^bright^) are shown in Figure 3. In lymph nodes, the majority of Treg cells expressed both Foxp3 and CD69, 55 (42–69) %, while in peripheral blood, only a minor fraction of Treg cells expressed both Foxp3 and CD69, 3.7 (2.6–6.7) %. The coexpression of Foxp3 and CD69 did not differ between SA and ACS patients.

The expression of IL-18R was used to identify the type 1 subsets of CD4^+^, CD8^+^ and NK cells in peripheral blood and lymph nodes [19], [20] (Table 4) (Figure S2). Type 1 CD4^+^ T cells appeared to be more frequent in blood and type 1 CD8^+^ T cells more frequent in lymph nodes while the percentages of type 1 NK cells were similar in blood and lymph nodes. However, certain differences in type 1 cells were noted when comparing SA and ACS patients. The differences between blood and lymph nodes reached significance only for type 1 CD4^+^ T cells in ACS patients and for type 1 CD8^+^ T cells in SA patients. The proportions of type 1 NK cells in blood were significantly increased in ACS patients compared with SA patients while no differences in type 1 NK cells was seen in lymph nodes.

**Table 4:** Distribution of type 1 lymphocyte subsets.

		SA patients (n = 13)	ACS patients (n = 13)	p
**IL-18R^+^ % of CD4^+^**	blood	4.9 (3.2–6.9)	6.3 (3.9–7.7)	ns
	lymph node	2.0 (1.5–4.6)	2.5 (1.8–4.8)*	ns
**IL-18R^+^ % of CD8^+^**	blood	3.4 (2.5–4.9)	5.3 (2.8–7.5)	ns
	lymph node	6.5 (4.9–15)**	5.9 (4.1–25)	ns
**IL-18R^+^ % of NK cells**	blood	3.4 (2.7–4.9)	7.3 (4.4–12)	<0.01
	lymph node	3.7 (2.7–5.5)	5.6 (2.1–9.1)	ns

Values are given as median (interquartile range).

Significant differences between patients with stable angina (SA) and acute coronary syndrome (ACS) were seen only in the IL^−^18R^+^ % of NK cell population in blood. Comparing blood and lymph nodes, there were significant differences in IL^−^18R^+^ % of CD4^+^ population for ACS and in IL^−^18R^+^ % of CD8^+^ population for SA. Values * p<0.05, ** p<0.01 refer to differences between lymph nodes and blood.

The expression of CCR4 was used to identify type 2 CD4^+^ T cells. In peripheral blood, the proportions of CCR4^+^CD4^+^ T cells were similar in SA and ACS patients, 14 (11–20) and 12 (9.0–19) %, respectively. In lymph nodes, the CCR4^+^CD4^+^ T cells were rare in both SA and ACS patients, 0.5 (0.2–0.7) and 0.5 (0.2–0.8) %, respectively (Figure S2).

The proportions of major lymphocyte subpopulations in peripheral blood did not show any significant correlations with the proportions of their respective counterparts in lymph nodes, except for CD8^+^ (*r* = 0.46, p<0.05). Among minor cell subpopulations, the percentages of HLA-DR+ CD8^+^ and CD4^+^Foxp3^+^ T cells in blood correlated weakly with their counterparts in lymph nodes (*r* = 0.48 and 0.40 respectively both p<0.05). In all subjects, a positive correlation was seen between CD4^+^CD69^+^ T cells and CD4^+^Foxp3^+^ T cells, *r* = 0.62, p<0.001) in peripheral blood, whereas no such correlation was seen in lymph nodes.

In order to confirm results in blood analyses, the percentages of major lymphocyte subpopulations were determined in a group of healthy controls (21 men, 6 women) in parallel with the patient samples. The distributions of cells were as follows (median and i-q range): CD3^+^ 64 (58–72) %, CD3^+^4^+^ 39 (32–47) %, CD3^+^8^+^ 21 (18–28) %, CD3^+^19^+^ 9.6 (7.0–14) %, and CD3^−^16/56^+^ (NK cells) 21 (13–31) %. Thus, all cell subpopulations in controls showed values similar to patients except for NK cells that were significantly more frequent in controls compared with ACS patients (p<0.05).

## Discussion

In the present study, we describe the lymphocyte distribution in thoracic lymph nodes of patients with multi-vessel coronary artery disease making a direct comparison with the corresponding populations in peripheral blood. The lymph nodes showed a cell composition that was substantially different from blood lymphocytes with a higher proportion of B cells, lower proportions of CD8^+^ T cells and NK cells and a 2-fold higher CD4/CD8 ratio. By ethical and practical reasons, the information on lymphocyte composition in lymph nodes in healthy individuals is sparse. However, our findings of lymphocyte distributions are in line with previous reports comparing blood and lymph node samples mainly obtained from patients with various types of cancer [21], [22], [23], [24], [25]. Further, our findings of low expression of the early activation marker CD69 in blood but substantially higher in lymph nodes is also in agreement with a previous study demonstrating the enrichment of CD69^+^ lymphocytes in both tumour reactive lymph nodes and control lymph nodes [25]. In contrast, the finding of similar proportions of HLA-DR expressing CD4^+^ and CD8^+^ T cells in blood and lymph nodes is not corroborated by earlier studies showing higher proportions of these cells in tumor lymph nodes than in blood [22], [24], findings that highlight the potential limitations of extrapolating data from peripheral blood in the diagnosis of immune activation.

Lymphocyte analyses in blood are widely used to evaluate the immune status in patients with various clinical presentations of coronary artery disease. There are several reports of enhanced systemic T cell activation, assessed by type 1 CD4^+^ T cells and IFN-gamma production, in ACS compared with SA patients [5], [6], [7], [8], [9], [10], [11] whereas a few others have shown similar levels of systemic T cell activation in both ACS and SA [12], [26]. According to Ranjbaran and coworkers [13], activation of the IFN-gamma axis was not associated with ACS but with a worse outcome at 1-year follow-up in both ACS and SA patients. In our study, there were no significant differences in CD4^+^ T cells, CD8^+^ T cells and activated T cell subsets between SA and ACS patients in either blood or lymph nodes. However, in ACS patients the proportions of IL-18R^+^CD4^+^ T cells were significantly higher in blood than in lymph nodes suggesting that an enhanced type 1 CD4^+^ T cell response in ACS patients may still exist in peripheral blood. The lack of major differences in immune status between SA and ACS may have several explanations. Since a median of 14 days passed between the ACS and surgery, it may be argued that an initial Th1 activation in ACS has declined when the myocardial injury is manifest. Another plausible explanation for the lack of difference between ACS and SA patients is that the T cell activation in ACS has become attenuated by statin therapy. While a majority of the ACS patients had received treatment less than 1 month it is known that Statin treatment exerts rapid immunomodulatory effects in ACS patients by inhibiting the Th1 response within a few days [27].

There is growing evidence that Treg cells exert atheroprotective effects through suppressive actions on Th1 polarization. Two previous studies have shown reduced numbers of circulating Treg cells in ACS compared with SA [6], [15]. However, in our study the proportions of Treg cells, defined as CD4^dim^25^bright^ or CD4^dim^25^bright^Foxp3^+^ T cells did not differ between SA and ACS in either blood or lymph nodes. Foxp3 was not only expressed in CD4^dim^25^bright^, i.e. strictly defined Treg cells [18], but also in CD25^dim^ CD4^+^ T cells, probably reflecting an activated T cell fraction. A transient expression of Foxp3 in human CD25^−^CD4^+^ T cells has been shown after in vitro stimulation, although without the gain of suppressive function [28]. Furthermore, we found that CD4^+^Foxp3^+^ T cells in blood but not in lymph nodes were strongly correlated with circulating CD4^+^CD69^+^ T cells supporting that they may represent a fraction of activated T cells in the circulation.

The only difference in immune status between SA and ACS involved NK cells that were significantly reduced in peripheral blood, but not in lymph nodes, of ACS patients. As expected, the CD56^dim^ NK cell fraction dominated over the CD56^bright^ NK cell fraction, in blood and to a lesser extent in lymph nodes [29]. In a previous study, we demonstrated that CD56^dim^ NK cells in blood were reduced in both SA and ACS patients compared with healthy individuals with the largest difference observed between ACS patients and controls [14]. We also reported that NK cells in patients were more prone to become activated and to undergo apoptosis ex vivo thus speculating that this could result in declined numbers of circulating NK cells [14], [30]. The discrepant results of NK cell proportions in SA patients and controls in our two studies may be explained by different time points of blood sampling and differences in clinical presentation; the present study comprising a more homogenous group with optimal anti-anginous medication. Nevertheless, the increased subfraction of circulating type 1 NK cells (IL-18R^+^) in ACS compared with SA indicates a more activated state of NK cells in the ACS patients. In vitro, the expression of IL-18R is associated with a dramatic increase in the production of type 1 cytokines like IFN-gamma [31], [32]. We have also recently seen that the plasma levels of IL-15, a cytokine with a distinct role in human NK cell activation [33], is increased in ACS patients (n = 20) compared with healthy controls (n = 37), (3.1 (2.7–3.6) vs 2.5 (2.2–3.2) pg/ml, p<0.05, (L. Jonasson, unpublished observations), a finding that may further support an increased activation state of NK cells in ACS. Still, we cannot exclude that the loss of circulating NK cells in ACS patients, due to activation or not, is merely the result of myocardial necrosis.

Lymphocytes are continuously moving between different compartments and a large amount of lymphocytes reside in lymph nodes. Therefore, a “snapshot” analysis of blood lymphocytes may not be representative of the current immune status. To our knowledge, this is the first study that makes a direct comparison between lymphocytes in lymph nodes and peripheral blood of patients with SA and ACS. By obvious reasons, the access to lymphoid tissue in patients with coronary artery disease was very limited not allowing us to include a larger number of patients or to collect more lymph nodes per patients. Based on the literature, upper mediastinal lymph nodes receive drainage from the heart whereas it is not clear from where internal mammary artery lymph nodes, used in this study, collect blood [34]. Thus, it is a potential limitation that the lymph nodes investigated may not directly be connected to the heart, although there are several connections across the highly dynamic lymph node network.

To summarize, we found several diversities in lymphocyte composition between lymph nodes and peripheral blood. However, the analysis of thoracic lymph nodes did not reveal any major changes in immune status between SA and ACS patients. The few significant changes observed in peripheral blood were not mirrored in lymph nodes. Although our results, due to the sample size, should be interpreted with caution, they indicate that lymph node analyses in multivessel coronary artery disease may not reveal any major changes in the immune response that are not detectable in peripheral blood.

## Supporting Information

Figure S1 **Gating strategies for activation markers.** Gating strategies for activation markers in peripheral blood and lymph node. T cells were defined by their CD3 expression (panel A) and then transferred for evaluation of the CD4 cells (panel B, CD69; panel C, HLA-DR) and CD8 cells (panel D, HLA-DR). Values are given as percent of CD4 and CD8, respectively. Examples from one ACS (acute coronary syndrome) patient shows dot plots representative for both SA (stable angina) and ACS patients.
(TIFF)

Figure S2 **Gating strategies for type 1 (IL-18 R^+^) and type 2 (CCR4^+^).** Gating strategies for type 1 (IL-18 R^+^) and type 2 (CCR4^+^) lymphocyte subsets in peripheral blood and lymph node. T cells were defined by their CD3 expression (panel A) and then transferred to panels B, C and E for evaluation of the CD4 and CD8 subsets, while CD56^+^ NK cells (panel D) were defined by their lack of CD3 expression in panel A. Values are given as percent of CD4, CD8 and CD56, respectively. Examples from one ACS (acute coronary syndrome) patient shows dot plots representative for both SA (stable angina) and ACS patients.
(TIF)

## References

[1] van der Wal AC, Piek JJ, de Boer OJ, Koch KT, Teeling P (1998). Recent activation of the plaque immune response in coronary lesions underlying acute coronary syndromes.. Heart.

[2] Hosono M, de Boer OJ, van der Wal AC, van der Loos CM, Teeling P (2003). Increased expression of T cell activation markers (CD25, CD26, CD40L and CD69) in atherectomy specimens of patients with unstable angina and acute myocardial infarction.. Atherosclerosis.

[3] Frostegård J, Ulfgren A-K, Nyberg P, Hedin U, Swedenborg J (1999). Cytokine expression in advanced human atherosclerotic plaques: dominance of pro-inflammatory (Th1) and macrophage-stimulating cytokines.. Atherosclerosis.

[4] Eid RE, Rao DA, Zhou J, Lo S-fL, Ranjbaran H (2009). Interleukin-17 and Interferon-{gamma} Are Produced Concomitantly by Human Coronary Artery-Infiltrating T Cells and Act Synergistically on Vascular Smooth Muscle Cells.. Circulation.

[5] Caligiuri G, Liuzzo G, Biasucci LM, Maseri A (1998). Immune system activation follows inflammation in unstable angina: pathogenetic implications.. Journal of the American College of Cardiology.

[6] Han S-F, Liu P, Zhang W, Bu L, Shen M (2007). The opposite-direction modulation of CD4+CD25+ Tregs and T helper 1 cells in acute coronary syndromes.. Clinical Immunology.

[7] Methe H, Brunner S, Wiegand D, Nabauer M, Koglin J (2005). Enhanced T-Helper-1 Lymphocyte Activation Patterns in Acute Coronary Syndromes.. Journal of the American College of Cardiology.

[8] Neri Serneri GG, Prisco D, Martini F, Gori A, Brunelli T (1997). Acute T-Cell Activation Is Detectable in Unstable Angina.. Circulation.

[9] Pasqui AL, Di Renzo M, Bova G, Maffei S, Pompella G (2006). Pro-inflammatory/anti-inflammatory cytokine imbalance in acute coronary syndromes.. Clin Exp Med.

[10] Patel KD, Duggan SP, Currid CA, Gallagher WM, McManus R (2009). High sensitivity cytokine detection in acute coronary syndrome reveals up-regulation of interferon gamma and interleukin-10 post myocardial infarction.. Clin Immunol.

[11] Steppich BA, Moog P, Matissek C, Wisniowski N, Kühle J (2007). Cytokine profiles and T cell function in acute coronary syndromes.. Atherosclerosis.

[12] Mazzone A, De Servi S, Vezzoli M, Fossati G, Mazzucchelli I (1999). Plasma levels of interleukin 2, 6, 10 and phenotypic characterization of circulating T lymphocytes in ischemic heart disease.. Atherosclerosis.

[13] Ranjbaran H, Sokol SI, Gallo A, Eid RE, Iakimov AO (2007). An Inflammatory Pathway of IFN-γ Production in Coronary Atherosclerosis.. The Journal of Immunology.

[14] Jonasson L, Backteman K, Ernerudh J (2005). Loss of natural killer cell activity in patients with coronary artery disease.. Atherosclerosis.

[15] Mor A, Luboshits G, Planer D, Keren G, George J (2006). Altered status of CD4+CD25+ regulatory T cells in patients with acute coronary syndromes.. European Heart Journal.

[16] Blum KS, Pabst R (2007). Lymphocyte numbers and subsets in the human blood: Do they mirror the situation in all organs?. Immunology Letters.

[17] Thygesen K, Alpert JS, White HD, Jaffe AS, Apple FS (2007). Universal definition of myocardial infarction.. Circulation.

[18] Mjösberg J, Svensson J, Johansson E, Hellström L, Casas R (2009). Systemic Reduction of Functionally Suppressive CD4dimCD25highFoxp3+ Tregs in Human Second Trimester Pregnancy Is Induced by Progesterone and 17β-Estradiol.. The Journal of Immunology.

[19] Borzychowski AM, Croy BA, Chan WL, Redman CWG, Sargent IL (2005). Changes in systemic type 1 and type 2 immunity in normal pregnancy and pre-eclampsia may be mediated by natural killer cells.. European Journal of Immunology.

[20] Chan WL, Pejnovic N, Lee CA, Al-Ali NA (2001). Human IL-18 receptor and ST2L are stable and selective markers for the respective type 1 and type 2 circulating lymphocytes.. J Immunol.

[21] Battaglia A, Ferrandina G, Buzzonetti A, Malinconico P, Legge F (2003). Lymphocyte populations in human lymph nodes. Alterations in CD4+ CD25+ T regulatory cell phenotype and T-cell receptor Vbeta repertoire.. Immunology.

[22] Morton BA, Ramey WG, Paderon H, Miller RE (1986). Monoclonal antibody-defined phenotypes of regional lymph node and peripheral blood lymphocyte subpopulations in early breast cancer.. Cancer Res.

[23] Santin AD, Ravaggi A, Bellone S, Pecorelli S, Cannon M (2001). Tumor-infiltrating lymphocytes contain higher numbers of type 1 cytokine expressors and DR+ T cells compared with lymphocytes from tumor draining lymph nodes and peripheral blood in patients with cancer of the uterine cervix.. Gynecol Oncol.

[24] Takenoyama M, Yasumoto K, Harada M, Matsuzaki G, Ishida T (1996). Expression of activation-related molecules on regional lymph node lymphocytes in human lung cancer.. Immunobiology.

[25] Vidal-Rubio B, Sanchez-Carril M, Oliver-Morales J, Gonzalez-Femandez A, Gambon-Deza F (2001). Changes in human lymphocyte subpopulations in tonsils and regional lymph nodes of human head and neck squamous carcinoma compared to control lymph nodes.. BMC Immunol.

[26] De Servi S, Mazzone A, Ricevuti G, Mazzucchelli I, Fossati G (1995). Clinical and angiographic correlates of leukocyte activation in unstable angina.. Journal of the American College of Cardiology.

[27] Link A, Ayadhi T, Bohm M, Nickenig G (2006). Rapid immunomodulation by rosuvastatin in patients with acute coronary syndrome.. Eur Heart J.

[28] Wang J, Ioan-Facsinay A, van der Voort EI, Huizinga TW, Toes RE (2007). Transient expression of FOXP3 in human activated nonregulatory CD4+ T cells.. Eur J Immunol.

[29] Fehniger TA, Cooper MA, Nuovo GJ, Cella M, Facchetti F (2003). CD56bright natural killer cells are present in human lymph nodes and are activated by T cell-derived IL-2: a potential new link between adaptive and innate immunity.. Blood.

[30] Li W, Lidebjer C, Yuan XM, Szymanowski A, Backteman K (2008). NK cell apoptosis in coronary artery disease: relation to oxidative stress.. Atherosclerosis.

[31] Fehniger TA, Carson WE, Caligiuri MA (1999). Costimulation of human natural killer cells is required for interferon gamma production.. Transplant Proc.

[32] Kunikata T, Torigoe K, Ushio S, Okura T, Ushio C (1998). Constitutive and induced IL-18 receptor expression by various peripheral blood cell subsets as determined by anti-hIL-18R monoclonal antibody.. Cell Immunol.

[33] Boyiadzis M, Memon S, Carson J, Allen K, Szczepanski MJ (2008). Up-regulation of NK cell activating receptors following allogeneic hematopoietic stem cell transplantation under a lymphodepleting reduced intensity regimen is associated with elevated IL-15 levels.. Biol Blood Marrow Transplant.

[34] Otsuka K, Terasaki F, Eishi Y, Shimomura H, Ogura Y (2007). Cardiac sarcoidosis underlies idiopathic dilated cardiomyopathy: importance of mediastinal lymphadenopathy in differential diagnosis.. Circ J.

