# Supplementary figures and images for: Lymphocyte Subpopulations in Lymph Nodes and Peripheral Blood: A Comparison between Patients with Stable Angina and Acute Coronary Syndrome
Source: PLoS One. 2012 Mar 1;7(3):e32691. doi: 10.1371/journal.pone.0032691 (PMC3291561; doi:10.1371/journal.pone.0032691)

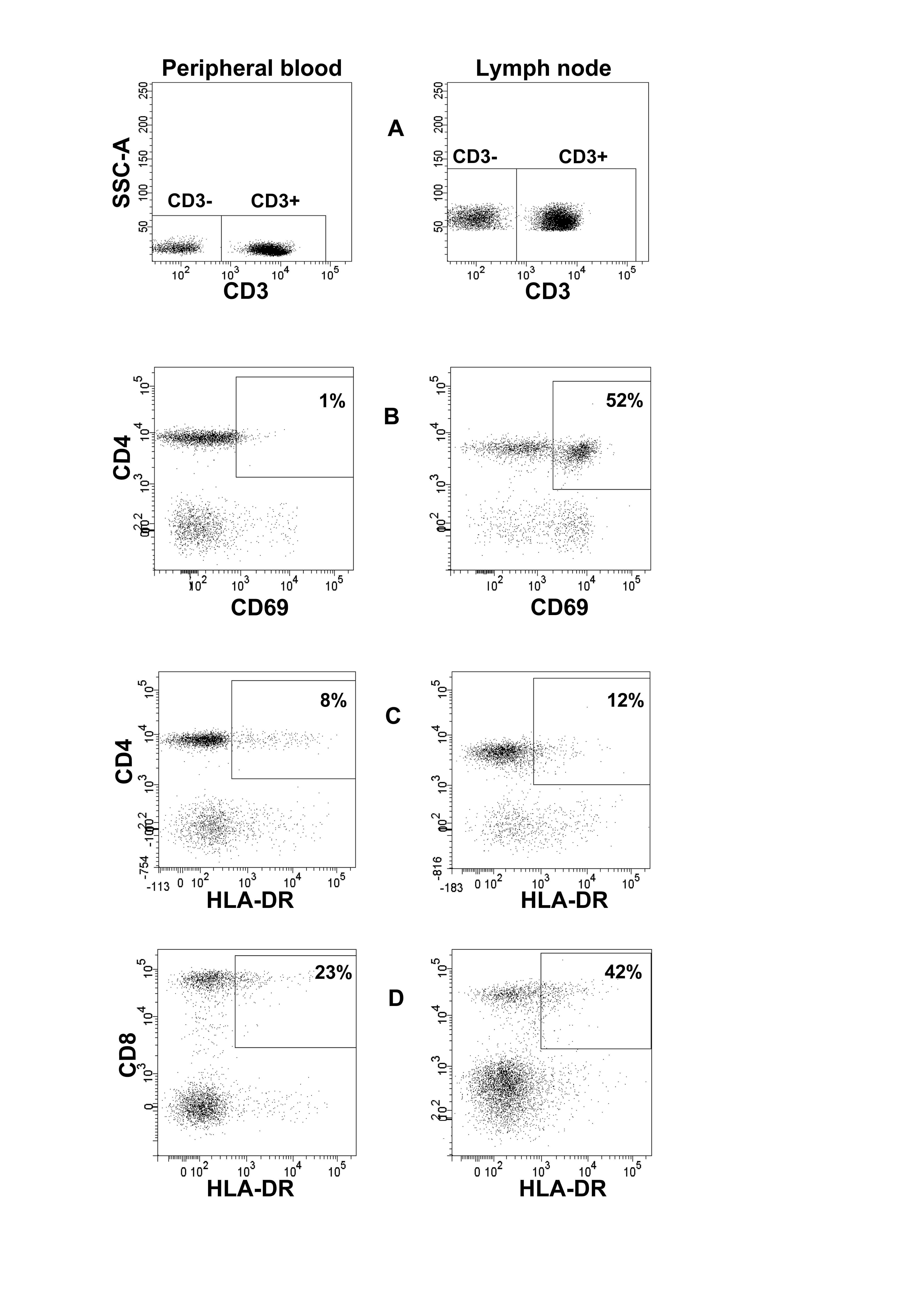

Supplement: Figure S1 — Gating strategies for activation markers. Gating strategies for activation markers in peripheral blood and lymph node. T cells were defined by their CD3 expression (panel A) and then transferred for evaluation of the CD4 cells (panel B, CD69; panel C, HLA-DR) and CD8 cells (panel D, HLA-DR). Values are given as percent of CD4 and CD8, respectively. Examples from one ACS (acute coronary syndrome) patient shows dot plots representative for both SA (stable angina) and ACS patients. (TIFF) [file pone.0032691.s001.tiff]

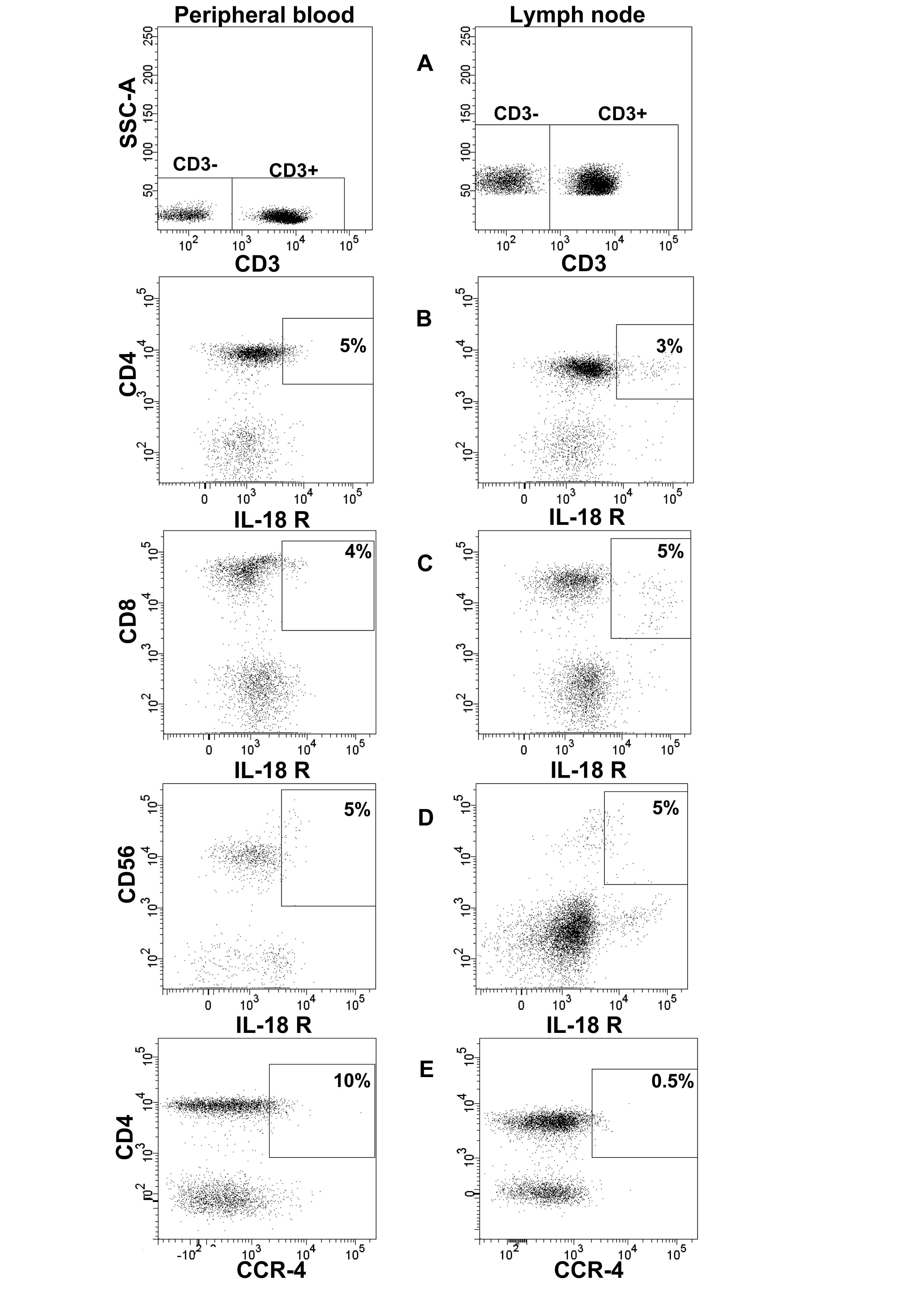

Supplement: Figure S2 — Gating strategies for type 1 (IL-18 R+) and type 2 (CCR4+). Gating strategies for type 1 (IL-18 R+) and type 2 (CCR4+) lymphocyte subsets in peripheral blood and lymph node. T cells were defined by their CD3 expression (panel A) and then transferred to panels B, C and E for evaluation of the CD4 and CD8 subsets, while CD56+ NK cells (panel D) were defined by their lack of CD3 expression in panel A. Values are given as percent of CD4, CD8 and CD56, respectively. Examples from one ACS (acute coronary syndrome) patient shows dot plots representative for both SA (stable angina) and ACS patients. (TIF) [file pone.0032691.s002.tif]
